# Three discipline collaborative radiation therapy (3DCRT) special debate: AI structure segmentation is *better* than clinician contouring for both OARs and targets

**DOI:** 10.1002/acm2.70183

**Published:** 2025-07-15

**Authors:** Andrew Hope, Michelle Mundis, Jan‐Jakob Sonke, John Kang, Stine Korreman, Brian Napolitano, Sharif Elguindi, Michael C. Joiner, Jay Burmeister, Michael M. Dominello

**Affiliations:** ^1^ Princess Margaret Cancer Centre University Health Network/Department of Radiation Oncology University of Toronto Toronto Ontario Canada; ^2^ Maryland Proton Treatment Center University of Maryland Baltimore Maryland USA; ^3^ Department of Oncology the Netherlands Cancer Institute Amsterdam Netherlands; ^4^ Department of Radiation Oncology and the Fred Hutch Cancer Center University of Washington Seattle Washington USA; ^5^ Danish Center for Particle Therapy, Department of Clinical Medicine Aarhus University Aarhus Denmark; ^6^ Department of Radiation Oncology Massachusetts General Hospital Boston Massachusetts USA; ^7^ Department of Radiation Oncology Memorial Sloan Kettering Cancer Center New York New York USA; ^8^ Department of Oncology Wayne State University School of Medicine Detroit Michigan USA; ^9^ Gershenson Radiation Oncology Center Barbara Ann Karmanos Cancer Institute Detroit Michigan USA

## 1 THREE DISCIPLINE COLLABORATIVE RADIATION THERAPY (3DCRT) SPECIAL DEBATE

Radiation Oncology is a highly multidisciplinary medical specialty, drawing significantly from three scientific disciplines—medicine, physics, and biology. As a result, discussion of controversies or changes in practice within radiation oncology involves input from all three disciplines, and sometimes more! For this reason, significant effort has been expended recently to foster collaborative multidisciplinary research in radiation oncology, with substantial demonstrated benefit. In light of these results, we endeavor here to adopt this “team‐science” approach to the traditional debates featured in this journal. This article is part of the series of special JACMP debates entitled “Three Discipline Collaborative Radiation Therapy (3DCRT)” in which each debate team typically includes a radiation oncologist, a medical physicist, and a radiobiologist. In this case, we have included a medical dosimetrist. We hope that this format will not only be engaging for the readership but will also foster further collaboration in the science and clinical practice of radiation oncology and developments thereof.

## 2 INTRODUCTION

Artificial intelligence (AI) is ubiquitous. The applications are limitless and the effects are permeative. The use of AI for contouring of organs at risk (OARs) has been in the works now for many years, however as algorithms have improved and adaptive replanning is becoming increasingly prevalent in the clinic, physicians and radiation oncology teams are increasingly reliant on software for auto contouring, including in certain scenarios for contouring targets. In this debate, we consider the risks and benefits of this progression towards increased contouring by AI. At what point does the machine definitively outperform the clinician? Are we there yet? For this debate we will argue exactly this point through the proposition, “AI structure segmentation is *better* than clinician contouring for both OARs and targets.” Arguing for the proposition will be John Kang, Stine Korreman, Brian Napolitano, and Sharif Elguindi. John Kang, MD, PhD, is an assistant professor in radiation oncology in the University of Washington Department of Radiation Oncology and the Fred Hutch Cancer Center. He is dual board certified in radiation oncology and clinical informatics and serves as clinical informatics lead. His clinical focus is on thoracic malignancies and his research focus is on natural language processing and informatics applications. Stine S Korreman, PhD, is Professor of Medical Physics at Aarhus University, Denmark. She leads a research group on AI for medical image analysis in radiotherapy with a focus on segmentation and dose prediction, and translation from research to clinical practice. She is chair of the ESTRO Focus Group AI in Radiotherapy and Director of the ESTRO course on AI in Radiotherapy. Brian Napolitano, MHL, CMD is Director of Medical Dosimetry at Massachusetts General Hospital in Boston, where he oversees treatment planning operations for photon and proton modalities at their main campus and satellite facilities. Brian is a former president of the American Association of Medical Dosimetrists (AAMD) and was the 2024 recipient of the AAMD Outstanding Achievement Award.  He received his Bachelor of Science degree in Biological Sciences from Binghamton University and his Master of Healthcare Leadership degree from Brown University.  Lastly, Sharif Elguindi, MS, DABR, is a Medical Physicist at Memorial Sloan Kettering Cancer Center where he serves as the AI clinical implementation lead. Together with his team, he has helped design, develop, and maintain an AI‐assisted contouring system that improves workflow efficiencies for over 10 000 treatments annually. His professional interests focus on designing and implementing software systems that support AI‐assisted target workflows for physicians.

Arguing against the propostion will be Andrew Hope, Michelle Mundis, and Jan‐Jakob Sonke. Andrew Hope, MD, FRCPC, is a Clinician Investigator in the Radiation Medicine Program, Princess Margaret Cancer Centre, Associate Professor in the Department of Radiation Oncology at University of Toronto and the Addie MacNaughton Chair in Thoracic Radiation Oncology. His research focuses on developing, deploying, and evaluating novel AI applications and other advanced technologies in the clinic. Michelle Mundis, MS, CMD, is a senior dosimetrist at Maryland Proton Treatment Center (MPTC). She has over 10 years of experience in radiation oncology, including roles as field service engineer, Varian Medical Systems, Medical Physics Assistant, and Clinical Coordinator for the University of Maryland Dosimetry Program. She currently serves as Secretary on the Board of Directors of the American Association of Medical Dosimetrists. Jan‐Jakob Sonke, PhD, leads a research group on adaptive radiotherapy at the Netherlands Cancer Institute (NKI). He is also the theme lead for image guided therapy at the NKI and full professor at the University of Amsterdam. He is one of the scientific directors of two labs focusing on the development of innovative AI algorithms for oncology and radiation therapy.

## 3 OPENING STATEMENTS

### 3.1 For the propostion (John Kang, Stine Korreman, Brian Napolitano, and Sharif Elguindi)

Segmentation in radiotherapy is essential for defining the location and shape of both targets and healthy tissues for treatment planning, impacting radiation dose distribution and treatment efficacy. Traditionally, this process has relied on the expertise of radiation oncologists, radiologists, physicists, dosimetrists, and therapists to manually delineate contours based on imaging, guidelines, and clinical judgment. However, manual segmentation is subject to inherent challenges including inter‐observer and intra‐observer variability, which can undermine treatment reproducibility and reliability and risk deviation from guidelines. Recent advances in AI‐assisted segmentation offer a promising solution, demonstrating the potential to reduce variability and enhance efficiency.

#### 3.1.1 Performance

Manual contouring, while central to the radiation therapy workflow, is inherently subjective. Variability arises from factors such as imaging resolution, interpretation of guidelines, and individual judgment, which are further influenced by training background, experience and cognitive factors like fatigue or bias. For example, some organ‐at‐risk (OAR) segmentations rely on international guidelines to standardize contours, yet interpretation varies widely among clinicians, introducing inter‐observer variability (IOV).^1^, ^2^ Similarly, clinical target volume segmentation is often based on consensus guidelines, while segmentation of gross tumor volume relies heavily on interpretation of images of limited resolution and variable contrast, leaving significant room for interpretation.^3^ The complexity of manual contouring is particularly evident in challenging cases with ambiguous tumor margins or limited soft‐tissue contrast, where precision is vital. Unfortunately, humans are inconsistent in their decision‐making, emphasizing the need for a more standardized approach.

AI assisted segmentation provides a robust tool to address these challenges. Once trained on sufficiently large and representative datasets within the domain specific tasks they are designed for, AI‐assisted segmentation can deliver highly consistent contours, minimizing the influence of subjective parameters such as cognitive fatigue and situational biases, and reducing inter‐ and intra‐observer variability,^4^ translating into more accurate treatment. Studies show that AI‐supported contours often align more closely with consensus guidelines and demonstrate fewer erroneous contours compared to manual efforts.^5^ Algorithms excel in the segmentation of complex structures where variability is common, enabling improved consistency that optimizes both treatment planning and delivery.^4^

#### 3.1.2 Efficiency

Contouring is a time‐intensive process; however, the time efficiency provided by AI‐assisted segmentation is significant.^6^ By automating segmentation, time is freed to allow clinicians to focus on treatment planning and patient interaction, helping to alleviate some of the workload pressures radiotherapy departments face. Notably, this aspect addresses the potential to alleviate the global care gap in radiotherapy by making high‐quality treatment more accessible in low‐resource settings. In many regions, shortages of trained oncologists and radiologists severely limit radiotherapy availability.^7^ AI‐assisted segmentation reduces dependency on local and highly specialized expertise and leverages models trained on global expertise to deliver consistent, guideline‐compliant care to underserved populations with limited resources. By automating much of the manual work involved in contouring, AI assisted segmentation can relieve time and workload pressures on clinicians in settings where both time and expertise are in short supply. This capability bridges disparities in cancer care across diverse resource settings, expanding access to effective radiotherapy for underserved populations.^8^

AI‐assisted segmentation is also crucial for implementing online adaptive radiotherapy which demands rapid re‐contouring while the patient is on the table. AI‐assisted segmentation enables clinicians to generate updated contours quickly, supporting frequent adjustments and more personalized treatments aligned with each patient's evolving anatomy and tumor characteristics.^9^, ^10^

#### 3.1.3 Continuous quality improvement

AI‐assisted segmentation continues to evolve, with many algorithms achieving high accuracy across various anatomical regions.^11^, ^12^ AI‐assisted segmentation is powered by machine learning frameworks that can be continuously improved by obtaining more training data. When humans edit target or OAR contours, these adjusted contours can then be compared with the AI‐contours to create bidirectional information flow. By utilizing advanced image recognition, multi‐modality inputs and large datasets, AI‐based systems can adapt to complex anatomical where high level of standardization and accuracy is challenging, if not impossible, to achieve through manual segmentation alone, even with extensive use of guidelines and training.

In conclusion, AI‐assisted segmentation addresses many limitations of manual contouring in treatment planning by offering advancements in performance, efficiency, and continuous quality improvement. By minimizing inter‐ and intra‐observer variability, AI‐assisted segmentation enhances the reproducibility of radiotherapy, contributing to safer, more effective, and more accessible patient care globally. Moreover, by supporting adaptive radiotherapy, AI‐assisted segmentation brings personalized cancer treatment closer to clinical reality. While human expertise remains essential for validating these contours, AI‐assisted segmentation represents a transformative advancement in precision, reliability, and equity in radiotherapy.

### 3.2 Against the proposition (Andrew Hope, Michelle Mundis, and Jan‐Jakob Sonke)

AI is developing rapidly and one of the applications with strong potential is the medical field and radiation oncology in particular.^13^ Most commonly, AI has been introduced into radiation clinics through the use of structure segmentation. Traditionally, dosimetrists and physicians would contour for OARs and targets manually, working together to ensure accuracy of these structures to precisely treat the tumor volumes and safeguard the surrounding OARs. Whilst many will agree that the implementation of auto segmentation has decreased contouring time and overall turnaround times for treatment planning and adaptation, the argument can be made that AI structure segmentation is currently not better than clinicians.

In terms of OAR contouring at the present time, novel AI structure segmentation based on foundation models may be superior to manual contouring.^14^ These algorithms, however, have a low technology ready level (TRL). Mature algorithms available in clinical practice may be more consistent and objective than clinician directed segmentation. Thus, the question changes from a question of consistency to a question of consistent accuracy. It can be seen that a structure delineated using AI, routinely does not start and stop at its true inferior and superior borders. This, for instance, creates consistent added work for the clinician to review and manually correct. Furthermore, while AI in general maintains objectivity, often subjectivity is needed in considering, for example, tumor invasion of the normal OARs. Contours created by AI often don't consider a patient's specific anatomical anomalies, such as aberrant vessels, inversions, or low frequency anatomic variants.^15^ Additionally, if contours are trained with a certain delineation atlas, they may not agree with clinical trial specific guidelines causing continued creation of manual contours. Due to the lack of high quality training data, auto‐segmentation performance for MRI‐guided RT is sub‐optimal. In all these instances, while efforts and time are saved by application of the auto segmentation, careful review and manual adjustments is still required.

Auto segmentation can also be utilized for target contouring.^16^ Currently available auto segmentation is only using the image‐based information during training and inference. Clinician‐based target contouring, however, relies not only on imaging information but also non‐imaging information such as physical examination findings and visual observations. In addition, a physician may increase or decrease clinical targets based on interpretation of tolerability of treatment. This requires a detailed understanding of patient comorbidities, age, chemotherapy use, disease state, and interactions of likely side effects with patient comorbidities, something AI's objectivity does not include. A physician's contouring strategy may include further knowledge of patient placement on neoadjuvant chemotherapy or during the course of adaptive radiotherapy meaning a different target volume. No database exists to train the AI or its learning model in these instances. Commercial companies do not have this information; only physicians working in multidisciplinary settings and communicating together maintain these standards. Training of AI models that do not account for these intrinsic variables will result in an “average” model which would under‐treat some patients and over‐treat others.

On a final note, AI structure segmentation may not be available for clinical use in most environments. While novel technology often makes a claim of reducing cost, this hypothetical benefit rarely materializes in practice. AI is currently being used within more academic or wealthier settings as auto segmentation has generally been provided as a separate package or license of its own. It often requires updates from the vendor which incur future additional costs to keep the technology up to date. This technology requires months of additional working hours for validation of the installation and updates, to training employees, etc. This can be problematic in an industry already facing budget cuts and financial hardships.

In conclusion, AI structure segmentation is an emerging technology with a number of potential applications but given all of the above noted limitations, it is not “better” than clinician contouring.

## 4 REBUTTAL

### 4.1 Rebuttal—For the propostion (John Kang, Stine Korreman, Brian Napolitano, and Sharif Elguindi)

While critics correctly note that AI‐generated contours require clinician review, with current guidelines mandating expert oversight for all AI segmentations,^17^ this review burden continues to diminish as models achieve greater accuracy. Recent advancements in AI architectures and training methodologies have dramatically improved performance metrics across anatomical regions. A comprehensive meta‐analysis demonstrated that AI segmentation not only achieves high contour accuracy but significantly reduces clinician workload, emphasizing that future gains primarily depend on refined training data and continued algorithm optimization.^18^ Even institutions with limited local datasets have successfully implemented transfer learning from larger public repositories while preserving model accuracy.^19^, ^20^ Thus, while human review remains an essential component of the workflow, the extent of manual editing required continues to decrease significantly.

Critics further argue that AI lacks the clinical judgment inherent in human expertise, however, recent approaches have successfully integrated clinical information into segmentation models. Advanced radiomics pipelines now extract quantitative imaging features linked to underlying pathology, while multimodal AI frameworks combine imaging data with relevant patient characteristics (including demographics and biomarkers) to guide contouring decisions. These methodologies have consistently demonstrated higher accuracy than traditional image‐only segmentation,^21^ effectively incorporating clinical context into the AI decision‐making process. Consequently, AI‐generated contours have the potential to increasingly align with expert clinical judgment while maintaining objectivity.

The concern that AI‐generated contours might lead to over‐ or under‐treatment paradoxically overlooks how standardized AI contouring often enhances treatment quality by eliminating human variability. In a retrospective analysis of the lung cancer trial RTOG 06–17, investigators found inconsistent manual heart contours may have led to significant underestimation of cardiac radiation dose when cases were retrospectively re‐contoured using AI.^22^ Therefore, rather than introducing risk of suboptimal treatment, AI contouring has the capacity to improve accuracy and ensure proper protection of critical structures.

Finally, regarding cost concerns, evidence clearly indicates that any initial investment is offset by substantial efficiency gains. The UK's National Institute for Health and Care Excellence (NICE) economic modeling provides clear break‐even thresholds based on contouring time saved, demonstrating the economic feasibility of AI implementation in clinical workflows.^23^ Empirical studies demonstrate that auto‐contouring reduces segmentation time by approximately 65% per target structure,^24^ easily exceeding these economic requirements. At institutional scale, this efficiency improvement frees clinician time for other critical tasks and offsets software acquisition costs. The significant time reduction represents both immediate workflow improvements and long‐term cost effectiveness.

Therefore, when considering overall productivity gains coupled with improved quality and consistency, AI segmentation represents not only a clinically valuable but also economically sound investment in radiotherapy planning.

### 4.2 Rebuttal—Against the propostion (Andrew Hope, Michelle Mundis, and Jan‐Jakob Sonke)

Our debate opponents have clearly described the limitations of manual contouring in radiation oncology in terms of inter and intra observer variability and the potential impact of AI segmentation in terms of improved consistency and efficiency. While we agree that AI segmentation has great potential, this is not the point of the debate. The question at hand is if AI segmentation is currently already outperforming clinicians.

The authors themselves state that AI segmentation requires training on “ … sufficiently large and representative datasets within the domain specific tasks they are designed for …”. In practice, however, current models are often based on single institution data sets as described by a recent review of Matoska et al.^11^ cited by the authors. Currently, it is challenging if not impossible to obtain sufficiently large multi‐institutional datasets to unlock the full potential of AI segmentation. Novel developments such as European Data Act^25^ and Federated Deep learning^26^ may extend the amount of data available for training improved models, but model performance with additional data requires evaluation.

The authors also highlight that “AI‐assisted segmentation is powered by machine learning frameworks that can be continuously improved by obtaining more training data.” While such continuous improvements are quite assessable in a research setting, there are currently insufficient validated methods of commissioning and validation of continuous learning in a clinical environment. Moreover, proposing the potential of continuous improvements also acknowledges sub‐optimal performance of currently available models.

Additionally, the authors acknowledge that “ … human expertise remains essential for validating these contours …”. This emphasizes, again, that AI segmentation currently does not provide super‐human performance but should be considered as a trainee that requires supervision. Current AI models make errors that humans would never make such as organ fragmentation or labeling organs in parts of the body where they do not occur. This demonstrates that current models lack a true understanding of the underlying anatomy.

In their argument, the authors mention that auto segmentation provides efficiency “… alleviating the global care gap … making high‐quality treatment more accessible in low resource settings.” As mentioned, models are often single institution based and require additional expertise, training and commissioning in order to let the AI perform to a clinically acceptable level locally, thus placing additional burden, “…on clinicians in settings where both time and expertise are in short supply.” The argued “…relieving time and workload pressures,” only occurs after months to years of work to implement. Furthermore, auto segmentation would not be an option in a low resource setting where technical and infrastructure limitations exist and where the additional cost is not feasible.

In summary, there may in the future be a performance advantage if a sufficient dataset is acquired however this is not the current state, and additional input from the oncology staff is required. Furthermore, additional time and effort is spent in implementing auto segmentation, currently diminishing any efficiency advances that are seen in centers that can afford the cost and time to implement the process.

## AUTHOR CONTRIBUTIONS

The first seven authors contributed equally to this work. All authors were responsible for preparation of arguments and in writing and reviewing the manuscript.

## CONFLICT OF INTEREST STATEMENT

Sharif Elguindi has two patents pending, one on an AI foundation model for 3D medical image segmentation and one for AI inference and quality assurance software.
